# Detection performance of PCR for *Legionella pneumophila* in environmental samples: a systematic review and meta-analysis

**DOI:** 10.1186/s12941-022-00503-9

**Published:** 2022-03-18

**Authors:** Xin Yin, Ying-Zhou Chen, Qi-Qing Ye, Li-Juan Liao, Zhuo-Rui Cai, Min Lin, Jia-Na Li, Geng-Biao Zhang, Xiao-Li Peng, Wen-Fang Shi, Xu-Guang Guo

**Affiliations:** 1 Department of Clinical Laboratory Medicine, The Third Affiliated Hospital of Guangzhou Medical University, Guangzhou, 510150 China; 2 Department of Pediatrics, The Pediatrics School of Guangzhou Medical University, Guangzhou, 510182 China; 3 Department of Clinical Medicine, The First Clinical School of Guangzhou Medical University, Guangzhou, 511436 China; 4 Department of Preventive Medicine, The School of Public Health of Guangzhou Medical University, Guangzhou, 511436 China; 5 Department of Traditional Chinese and Western Medicine in Clinical Medicine, The Clinical School of Traditional Chinese and Western Medicine of Guangzhou Medical University, Guangzhou, 511436 China; 6 Department of Medical Imaging, The Second Clinical School of Guangzhou Medical University, Guangzhou, 511436 China; 7 Department of Clinical Medicine, The Third Clinical School of Guangzhou Medical University, Guangzhou, 511436 China; 8 Key Laboratory for Major Obstetric Diseases of Guangdong Province, The Third Affiliated Hospital of Guangzhou Medical University, Guangzhou, 510150 China; 9 Key Laboratory of Reproduction and Genetics of Guangdong Higher Education Institutes, The Third Affiliated Hospital of Guangzhou Medical University, Guangzhou, 510150 China

**Keywords:** *Legionella pneumophila*, *L. pneumophila*, PCR, Diagnosis

## Abstract

**Background:**

Legionellosis remains a public health problem. The most common diagnostic method to detect *Legionella pneumophila* (*L. pneumophila*) is culture. Polymerase chain reaction (PCR) is a fast and accurate method for this detection in environmental samples.

**Methods:**

Four databases were searched for studies that evaluated the detection efficiency of PCR in *L. pneumophila*. The quality evaluation was conducted using Review Manager 5.3. We used Meta-DiSc 1.4 software and the Stata 15.0 software to create forest plots, a meta-regression, a bivariate boxplot and a Deeks’ funnel plot.

**Results:**

A total of 18 four-fold tables from 16 studies were analysed. The overall pooled sensitivity and specificity of PCR was 94% and 72%, respectively. The positive likelihood ratio (RLR) and negative likelihood ratio (NLR) was 2.73 and 0.12, respectively. The result of the diagnostic odds ratio (DOR) was 22.85 and the area under the curve (AUC) was 0.7884.

**Conclusion:**

Establishing a laboratory diagnostic tool for *L. pneumophila* detection is important for epidemiological studies. In this work, PCR demonstrated a promising diagnostic accuracy for *L. pneumophila*.

**Supplementary Information:**

The online version contains supplementary material available at 10.1186/s12941-022-00503-9.

## Background

*Legionella* is a Gram-negative bacterium that thrives in warm and humid environments [1]. It is difficult to control owing to its resistance to disinfectants, especially in artificial aquatic environments [2].Currently, the genus* Legionella* includes more than 58 species and 70 distinct serogroups. All species are susceptible to legionellosis, among which *Legionella pneumophila* (*L. pneumophila*) serogroup 1 is the most prevalent pathogenic bacterium [3]. Legionellosis may manifest as Pontiac fever, which is characterized by respiratory flu, and Legionnaires’ disease which is characterized by a serious lung infection and multisystem damage. Pontiac fever is a self-limited febrile illness, whereas Legionnaires’ disease is a severe form of pneumonia with a high fatality rate [4].

Legionellosis is caused by the inhalation of aerosols contaminated with *Legionella* spp. including *L. pneumophila* observed in artificial water sources such as hot tubs, cooling towers, showers, air conditioning and plumbing systems [1]. Approximately 90% of the diseases caused by *Legionella* can be prevented by better water control [5]. Therefore, it is crucial to rapidly assess the number of live or dead microbes present in water samples for public health, especially in high-risk environments such as hospitals and nursing homes. [6]. To reduce the mortality of legionellosis, it is necessary to develop an effective and rapid method to detect *Legionella*, especially *L. pneumophila* from environmental sources [7].

Currently, there are two main ways to detect *L. pneumophila* which are the culture and polymerase chain reaction (PCR) methods [8]. Although the agar plate culture has long been considered the gold standard for detecting *L. pneumophila* from primary samples, it does have inevitable limitations [9]. First, it takes 10–14 days to have visible *L. pneumophila* colonies [10]. Second, it requires both hard work and professional skills to identify *L. pneumophila* correctly. Differences in test conditions and technique may influence the results [11]. Moreover, other microorganisms in the specimen may inhibit the growth of *L. pneumophila*, resulting in false-negative results [12]. In contrast, PCR is a faster, easier and more accurate method to detect *L. pneumophila* in environmental samples, which is also applicable to large-scale detections [13]. Furthermore, on-site PCR allows for robust and straightforward quantification of *L. pneumophila* species in the field for routine monitoring, rapid response and effective control of infectious outbreaks [14].

Considering this situation, we conducted this study and evaluated the efficiency of PCR for *L. pneumophila* according to Preferred Reporting Items for Systematic Reviews and Meta-Analyses (PRISMA) diagnostic test accuracy guidelines.

## Methods and materials

Patients were not involved in this study. Therefore, institutional review body permission was not required. All our review processes adhered to the PRISMA statement guidelines (http://www.prisma-statement.org/).

### Search strategy

Articles about *Legionella* and PCR were systematically searched for by two reviewers. All data were available in PubMed, Embase, Web of Science, Cochrane Library, WanFang, China National Knowledge Infrastructure and Chinese Biomedical Literature database before February 2021. The keywords ‘PCR, Polymerase Chain Reactions’ and ‘*Legionella*’ were used for the advanced search (see Additional file 1). Geographical restrictions were not applied in these articles.

### Screening criteria of included studies

Two researchers independently screened the title/abstract, followed by the full text, using predetermined reviewing criteria designed by the third reviewer. The final decision was made by the third reviewer when there was a dispute between two reviewers.

The inclusion criteria were as follows: (1) PCR was the detection method; (2) *Legionella* was detected; (3) environmental samples were included; (4) the study was original research and related to diagnostics.

The exclusion criteria were as follows: (1) duplicate studies; (2) culture was not the gold standard; (3) case reports, conference summaries, reviews and editorials; (4) visual four-fold tables; (5) sample size < 20; (6) *Legionella* spp. without *L. pneumophila*; (7) the language was not English.

### Data extraction

The investigators carefully read the included articles. Relevant data were extracted from the studies, including publication information (e.g., the first author, publication time, country, sample source, PCR type and targeted gene), and arranged made into 2 × 2 fourfold tables filled with true-positive (TP), true-negative (TN), false-positive (FP), and false-negative (FN) results. Investigators independently extracted the data. When there was a discrepancy in the extracted data, the two investigators in charge discussed and decided, or the third investigator was asked.

### Quality assessment

Two review authors independently assessed of the risk of bias to evaluate the quality, using the Quality Assessment of Diagnosis Accuracy Studies-2 (QUADAS-2) guidelines [15]. The risk of bias in each part was rated ‘high’, ‘unclear’, or ‘low’. Differences were resolved through discussion with the third reviewer. The quality figures were created by the Review Manager version 5.3.

### Statistical analysis

We obtained the numbers of TP, FP, FN and TN results from each enrolled study. Using a random-effects model, we calculated the following indicators of detection accuracy: sensitivity, specificity, positive likelihood ratio (PLR), negative likelihood ratio (NLR), diagnostic odds ratio (DOR) and their 95% confidence intervals (CIs). The subject operating characteristic (SROC) curve and the area under the SROC curve (AUC) were used to summarise the overall test performance.

Heterogeneity was identified from a threshold effect with the P value of Spearman correlation. Meanwhile, the non-threshold effect was assessed based on DOR. We conducted a meta-regression analysis and generated a bivariate box plot to evaluate the outliers and describe the diagnosis value. Publication bias was tested using Deeks’ funnel plot asymmetry. P value < 0.05 was considered statistically significant. MetaDiSc 1.4 and Stata 15.0 were used to analyse the results.

## Results

### Eligible studies and characteristics

A total of 7951 publications were retrieved based on the search strategy. After we eliminated the repetitive items, 3872 articles remained. For their uncorrelated titles or abstracts, 3809 studies were removed. Following a thorough review of 63 studies, 47 articles were excluded for various reasons (see Additional file 2). Finally, 16 qualified articles were included [7, 9, 13, 16–28]. A total of 18 fourfold tables were extracted from these included articles. The characteristics of the studies included were presented in Table 1.

**Table 1:** Characteristics of the included studies (N = 16)

Author	Year	Country	Sample source	TP	FP	FN	TN	Total	PCR type	Gene
Catalan	1994	Spain	Hospital room (cold water and hot water)	12	7	0	15	34	Nested PCR	*mip*
Fricker	1995	UK	–	33	24	8	12	77	PCR	*mip*
Fiume (a)	2005	Italy	Hospitals and private habitations	77	11	0	36	124	Nested PCR	*mip*
Fiume (b)	2005	Italy	Hospitals and private habitations	72	9	5	38	124	PCR	*mip*
Yaradou	2007	France	Water distribution system, cooling tower	65	50	9	54	178	r-qPCR	–
Behets	2007	Belgium	Power plants cooling circuits, tap water	10	4	0	16	30	r-qPCR	–
Yáñez	2007	Spain	Citical points and cooling tower samples	35	14	0	6	55	Seminested PCR	*dotA*
Morio	2007	France	6 distinct sites at hospital	27	30	4	59	120	r-qPCR	*mip*
Bonetta	2009	Italy	Cold water at hotel inlet, hot water from boiler, room showers and recycling	19	18	0	39	76	r-qPCR	*mip*
Fittipaldi (a)	2010	Terrassa	Cooling tower or hot tap water	19	10	3	18	50	r-qPCR	*dot*
Fittipaldi (b)	2010	Terrassa	Big buildings	21	8	1	20	50	r-qPCR	*mip*
Lee	2011	UK	Cooling tower, domestic water, spa pools and hot tubs	311	220	13	193	737	qPCR	–
Al-Matawah	2012	Kuwait	Wash basins and showerheads in bathrooms, taps and tanks from kitchens	45	41	4	114	204	rRT-PCR	–
Grúas	2014	Spain	Terminal points of water network	12	14	6	13	45	rRT-PCR	*mip*
Collins	2015	UK	Surface water, water systems, etc.	31	13	0	156	200	r-qPCR	*mip*
Tabatabaei	2016	Iran	Hospitals, educational departments, shopping centers, etc.	4	10	0	20	34	PCR	*icmO* and *sidA* and *lidA*
Collins	2017	UK	Cooling towers, spa pools, ship waters	181	383	5	1433	2002	r-qPCR	–
Toplitsch	2018	Austria	Drinking water, cooling towers or water	28	24	6	25	83	qPCR	*Mip*

*mip,* *macrophage infectivity potentiator*; *dot,* defective organelle trafficking; *icmO*, *sidA* and *lidA* are *Legionella*-specific virulence determinants

### Quality assessment

**Fig. 1:**
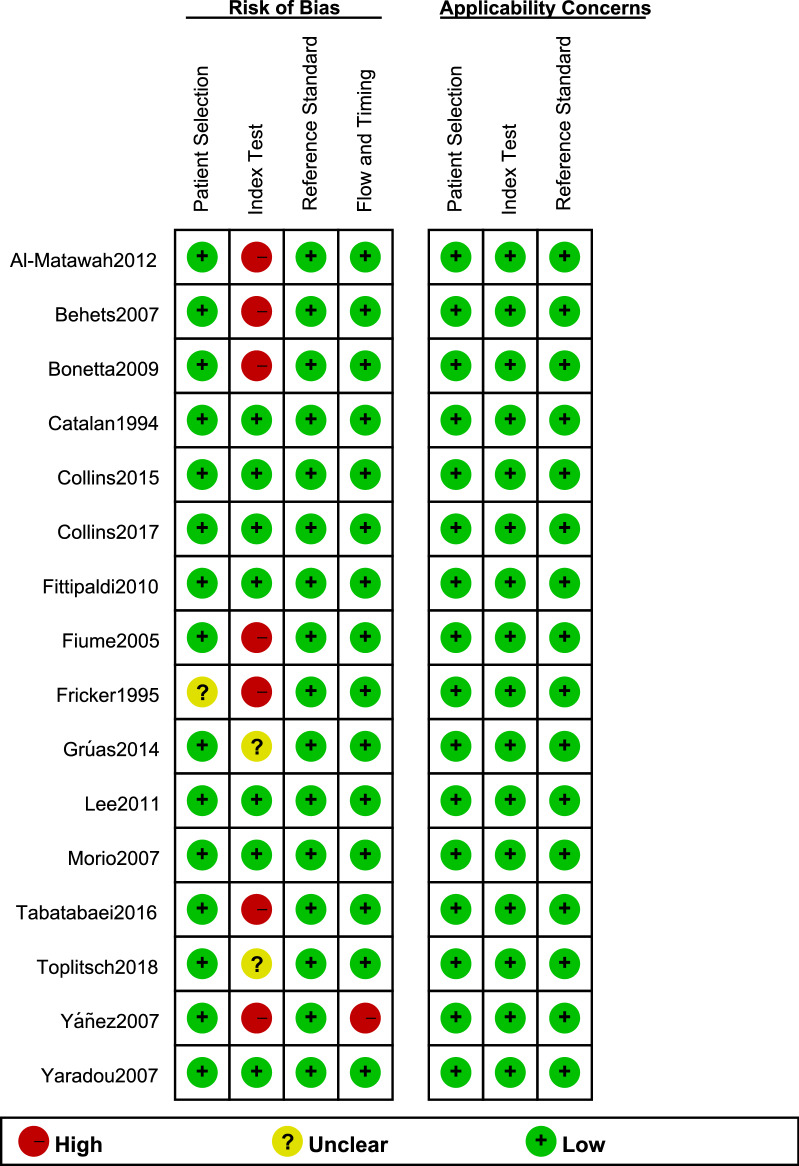
Methodological quality summary of included studies

**Fig. 2:**
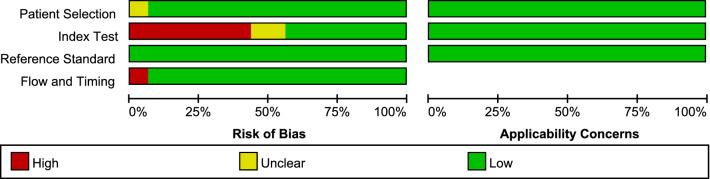
Methodological quality graph of included studies

The overall quality of the 16 included studies was shown in Figs. 1 and 2. Considering that the thresholds of the index employed were not predetermined, seven studies (43.75%) were at high risk of bias in the index test domain. Only one study (6.25%) was rated as ‘high risk’ in the flow and timing domain because not all cases were included in the analysis.

### Results of PCR

**Fig. 3:**
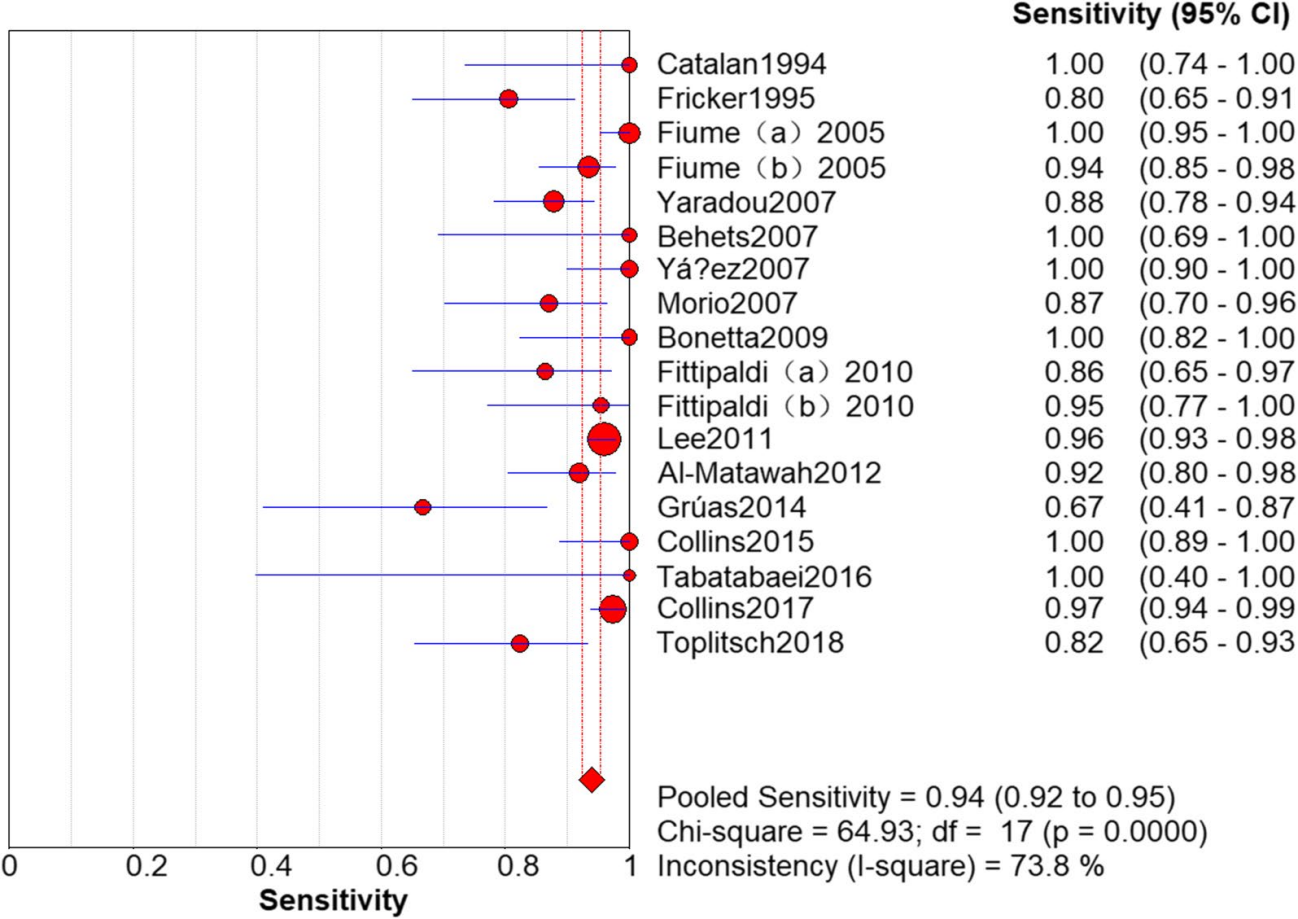
Sensitivity of polymerase chain reaction (PCR) in the detection of *L. pneumophila*

**Fig. 4:**
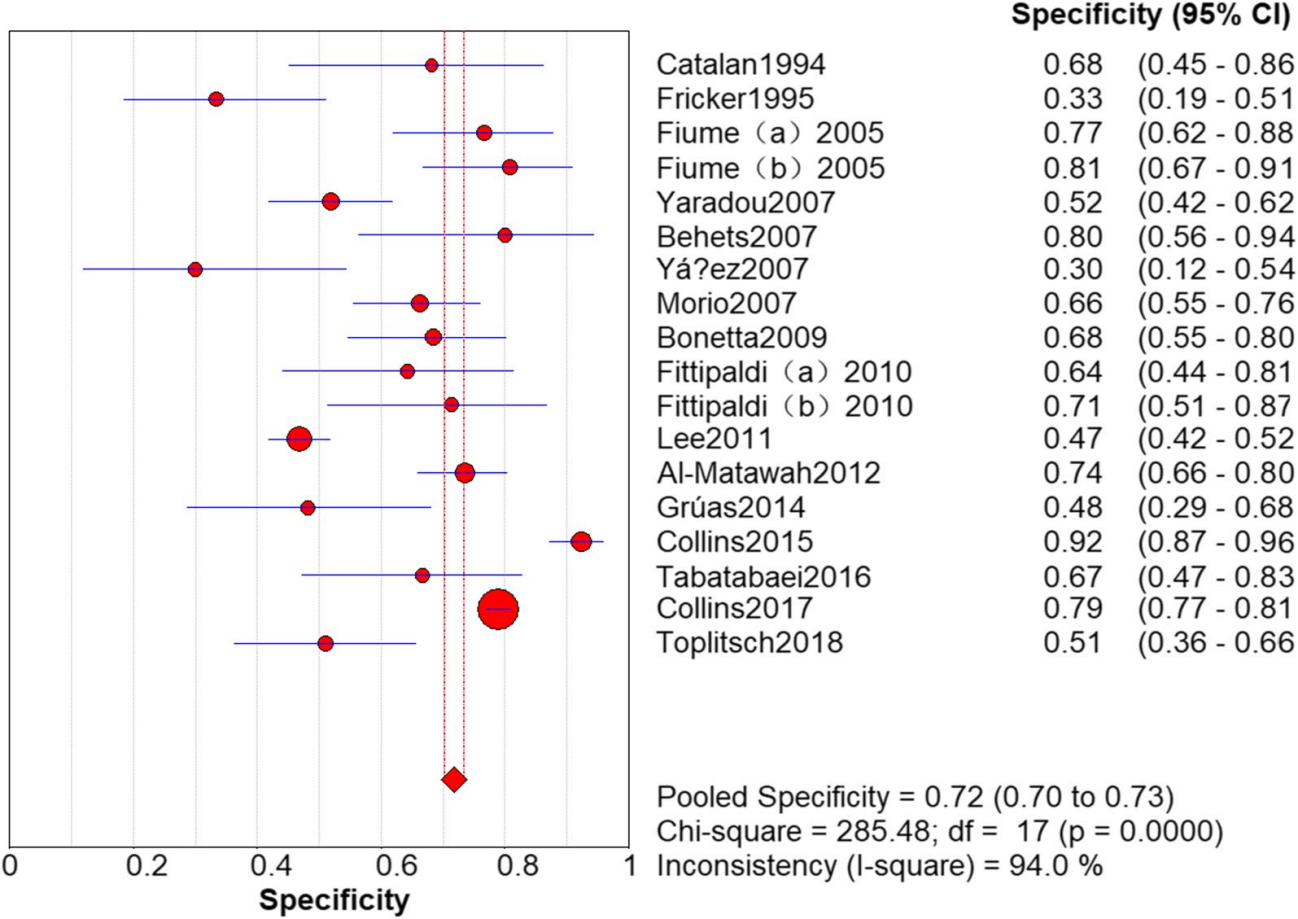
Specificity of polymerase chain reaction (PCR) in the detection of *L. pneumophila*

**Fig. 5:**
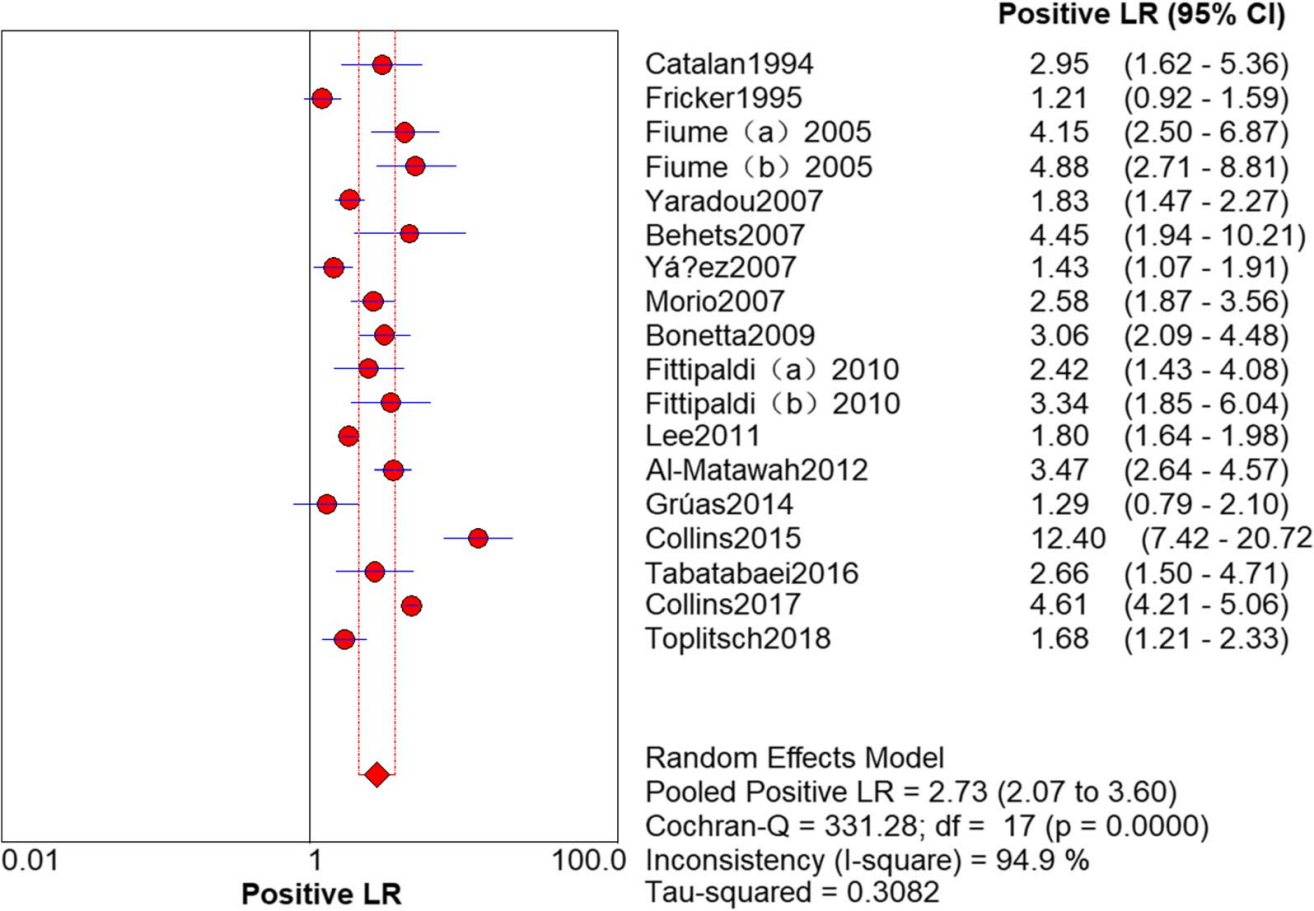
Positive likelihood ratio (PLR) of polymerase chain reaction (PCR) in the detection of *L. pneumophila*

**Fig. 6:**
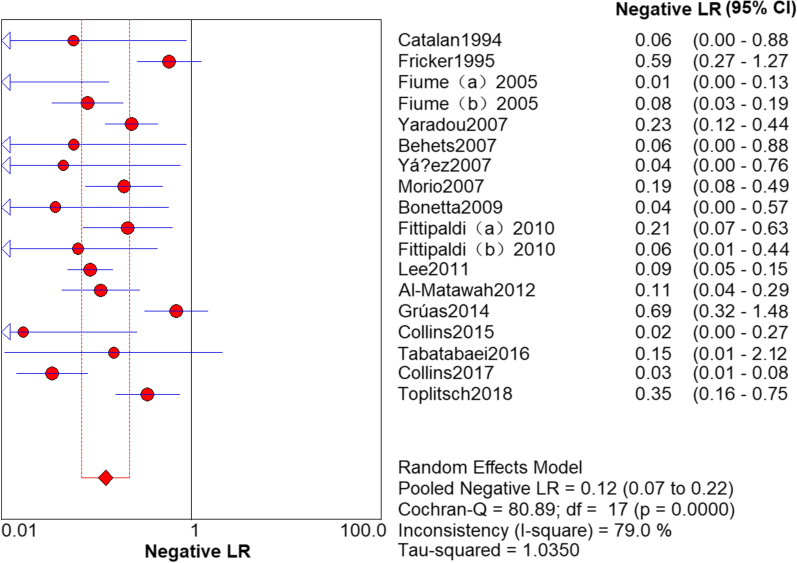
Negative likelihood ratio (NLR) of polymerase chain reaction (PCR) in the detection of *L. pneumophila*

**Fig. 7:**
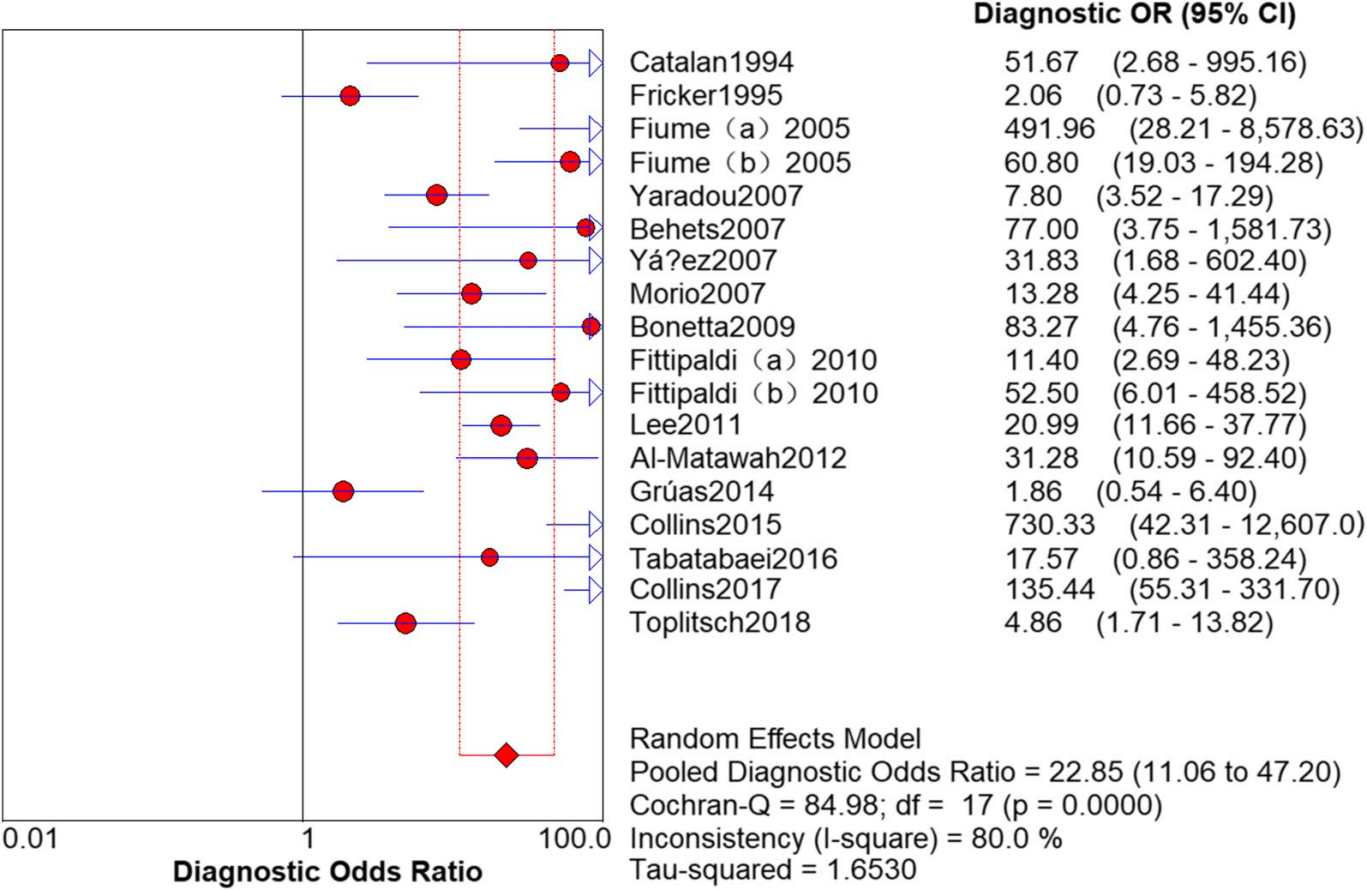
DOR of PCR in the detection of *L. pneumophila*

The sensitivity and specificity of PCR in the detection of *L. pneumophila* was 0.94 (95% CI 0.92–0.95) and 0.72 (95% CI 0.70–0.73), respectively (Figs. 3 and 4). The PLR and NLR was 2.73 (95% CI 2.07–3.60) and 0.12 (95% CI 0.07–0.22), respectively (Figs. 5 and 6). The DOR was 22.85 (95% CI 11.06–47.20) in Fig. 7.

### Threshold effect analysis

**Fig. 8:**
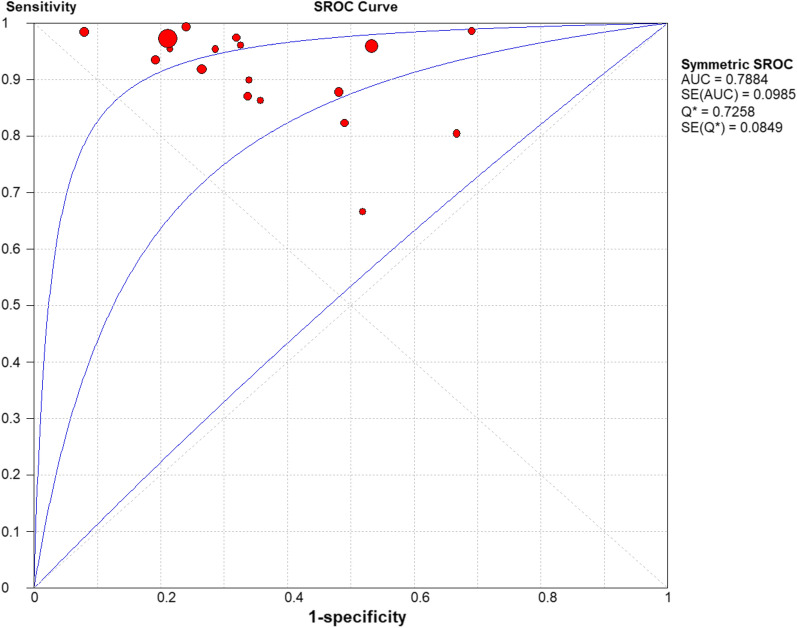
Summary receiver operating characteristic curve

It can be observed from the SROC curve (Fig. 8) that there was no ‘shoulder-arm’ distribution. In addition, the Spearman correlation coefficient was -0.446 (< 0.6), and the P value was 0.064 (> 0.05) (see Additional file 3). The automatically generated I-square (I^2^) was interpreted that 50–90% represents substantial heterogeneity, and 75–100% means considerable heterogeneity [29]. Therefore, we concluded that it was not a threshold effect. High heterogeneity was detected as follows (Figs. 3, 4, 5, 6, 7): sensitivity, I^2^ = 73.8%; specificity, I^2^ = 94.0%; PLR, I^2^ = 94.9%; NLR, I^2^ = 79.0% and DOR, I^2^ = 80.0%.

### SROC curve

The AUC was 0.7884 in the SROC curve. These indicated a considerable diagnostic accuracy of PCR for *L. pneumophila* (Fig. 8).

### Meta-regression analysis and bivariate box plot

**Fig. 9:**
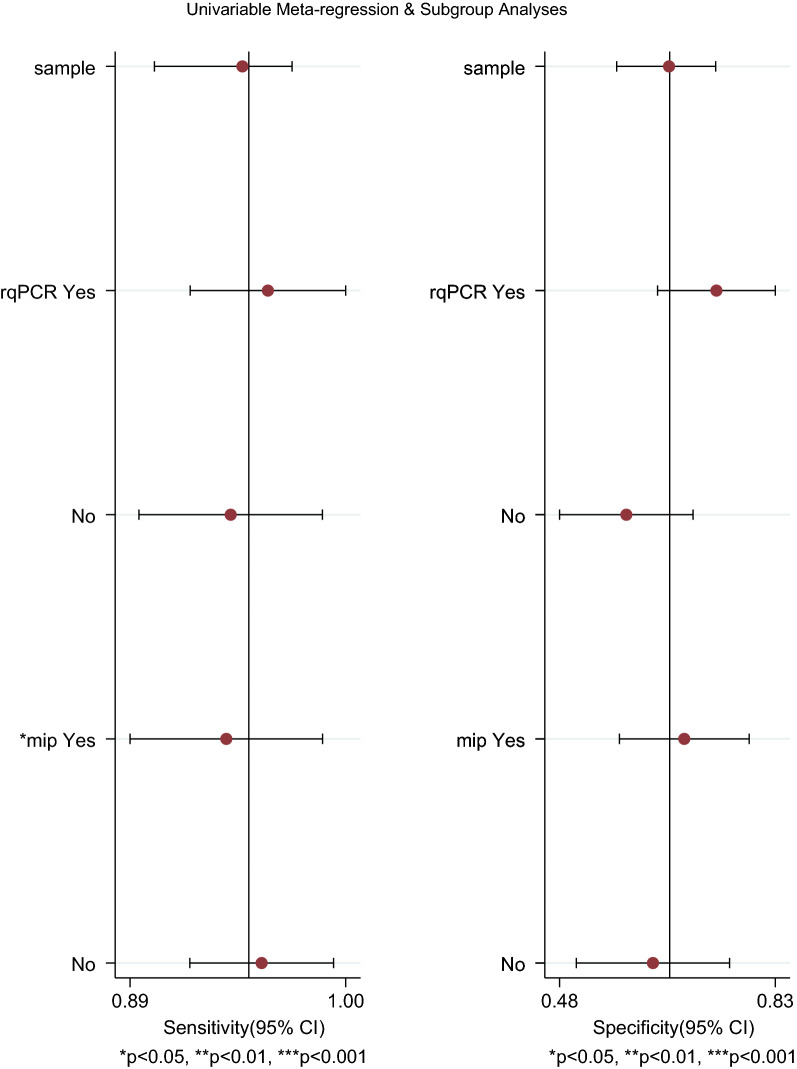
Meta-regression for heterogeneity analysis

**Fig. 10:**
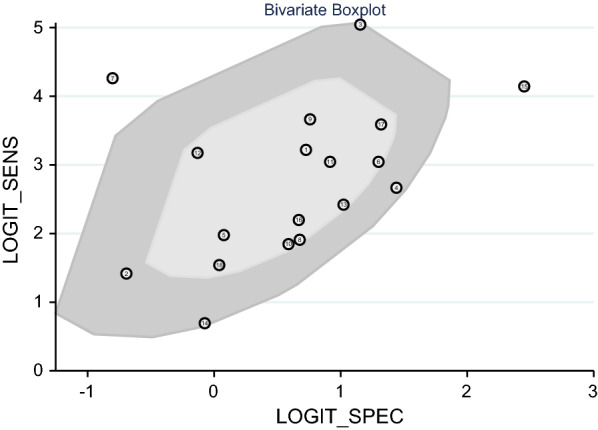
Bivariate box plot for heterogeneity analysis

A statistical association with sensitivity was observed in the *macrophage infectivity potentiator *(*mip*) gene (P < 0.05) in Fig. 9. For the bivariate boxplot in Fig. 10, two floating points were out of the circles suggesting heterogeneity [9, 20].

### Publication bias

**Fig. 11:**
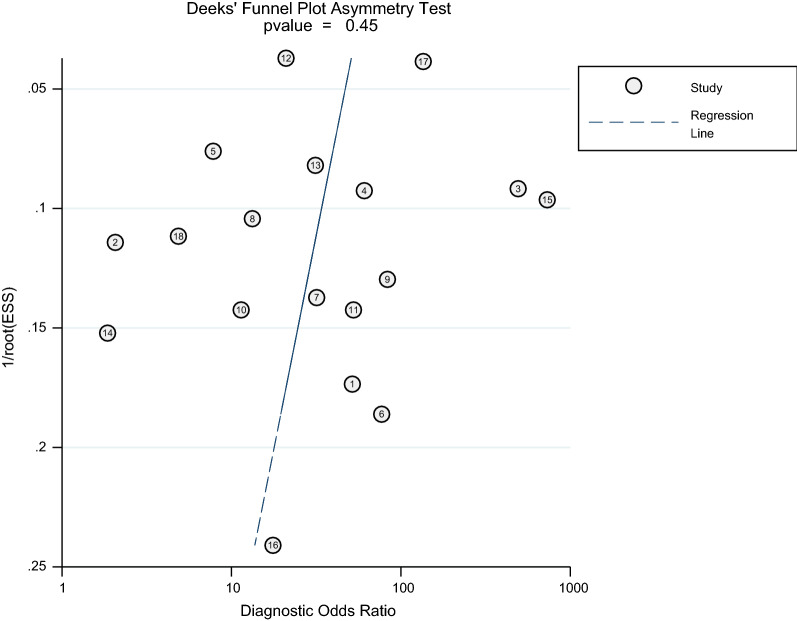
Deeks’ funnel plot asymmetry test

In Deeks’ funnel plot (Fig. 11), most points were distributed symmetrically along both sides, and the P value was 0.45 (> 0.1), indicating no publication bias in the study.

## Discussion

*L. pneumophila*, the most important causative agent of legionellosis, is a harmful pathogen that is often found in water systems [30, 31]. The overall case-fatality rate of legionellosis is 5–14%, but 76% when inappropriate antibiotics were used [32]. Therefore, it is of great importance to establish a standardised method for early and rapid environmental detection of *L. pneumophila* to prevent outbreaks of infection in hospitals.

Culture has been recognised as the gold standard for the detection of *L. pneumophila*; however, it is not widely used in environmental detection because it is time-consuming and limited by the culture process which is affected by other rapid propagation strains [6]. As an alternative method, PCR provides a faster turnabout time, a higher level of sensitivity and the possibility of early rapid detection. However, compared with the culture method, the cost is higher. In addition, there may be false-positive results when the amount of *L. pneumophila* is very small and not pathogenic [33].

Our analysis revealed a sensitivity of 0.94 (95% CI 0.92–0.95) and a relatively low specificity of 0.72 (95% CI 0.70–0.73) for PCR detection of *L. pneumophila*. The reason for the low specificity may be that PCR analyses amplified DNA in environmental samples, including DNA from dead bacteria and living bacteria that cannot be cultured. Moreover, when environmental samples are cultured, *L. pneumophila* can be inhibited by other overgrown bacteria owing to its specific growth requirements. Furthermore, *L. pneumophila* cells may reduce since the acid buffer or heat treatment is used in the sample preparation [20, 23, 25]. Dead bacteria can be detected by PCR, which will result in false-positive results. The presence of PCR inhibitors in water samples can lead to false-negative results [21].

In terms of heterogeneity, it was concluded that high heterogeneity was caused by the non-threshold effect rather than the threshold effect. The *mip* gene indicated potential heterogeneity in sensitivity analysis but not in specificity analysis. Moreover, subgroup analysis was not conducted owing to insufficient samples, although samples were obtained from different sources such as cooling towers, ship water and diverse water supply systems. [26–28].

Different temperatures, acid–base environments and disinfection conditions can influence the growth of *L. pneumophila* in water samples, resulting in heterogeneity. Furthermore, PCR cannot distinguish between dead bacteria and live bacteria, therefore, diverse water samples may exhibit different specificities to PCR, as described previously [34]. In addition, we speculate that the test inspectors and experimental conditions may have contributed to heterogeneity.

In this study, we found two studies exhibited substantial heterogeneity, according to the bivariate boxplot. When PCR was performed on the contaminated samples including dead bacteria and bacteria with low viability, it resulted in a higher sensitivity and a lower specificity [9, 20]. To quantify the viable bacteria before PCR, DNA was treated with ethidium or propidium monoazide for amplification, which improved sensitivity [9].

However, our study had some limitations. First, the disagreement between the two reviewers on included studies and extracted data was resolved, but it cannot be quantified with Cohen's Kappa score and introduced selection bias. Second, exclusion of grey literature and non-English studies could introduce selection bias. Last, the results may be influence by the different primers and probes used.

## Conclusions

Inconclusion, PCR has been considered beneficial for *L. pneumophila* in environmental samples owing to its rapid turn-around time and high sensitivity, and the ability to detect small amounts of target nucleic acids in samples. The results have proven to be crucial for environmental public health, especially for environmental surveillance in hospitals and large water systems. PCR may enable prevention and early diagnosis of Legionellosis. Therefore, efficient and convenient PCR may be a major laboratory diagnostic tool for epidemic prevention of Legionellosis.

## Supplementary Information

**Additional file 1: Table S1.** Literature search strategy.

**Additional file 2: Figure S1.** Flow diagram of inclusion and exclusion.

**Additional file 3: Table S2.** Analysis of diagnostic threshold.

## Data Availability

Not applicable.

## Abbreviations

CI: Confidence interval
AUC: Area under the curve
DOR: Diagnosis odds ratio
Fig: Figure
FN: False negative
FP: False positive
mip: *Macrophage infectivity potentiator*
NLR: Negative likelihood ratio
PCR: Polymerase chain reaction
PLR: Positive likelihood ratio
PRISMA: Preferred reporting items for systematic reviews and meta-analyses
QUADAS: Quality Assessment of Diagnostic Accuracy Studies
SROC: Summary receiver operating characteristic
TN: True negative
TP: True positive
