# Supplementary material for: Detection performance of PCR for Legionella pneumophila in environmental samples: a systematic review and meta-analysis
Source: Ann Clin Microbiol Antimicrob. 2022 Mar 18;21:12. doi: 10.1186/s12941-022-00503-9 (PMC8934000; doi:10.1186/s12941-022-00503-9)
Supplement: Supplementary file 1 — Additional file 1: Table S1. Literature search strategy. [file 12941_2022_503_MOESM1_ESM.docx]

Additional file 1. Literature search strategy

| Search  Number | Description |
| --- | --- |
| 1 | ‘PCR’ OR ‘Polymerase Chain Reactions’ OR ‘Reaction, Polymerase Chain’ OR ‘Reactions, Polymerase Chain’ OR ‘Inverse PCR’ OR ‘PCR, Inverse’ OR ‘Inverse Polymerase Chain Reaction’ OR ‘Nested Polymerase Chain Reaction’ OR ‘Nested PCR’ OR ‘PCR, Nested’ OR ‘Anchored PCR’ OR ‘PCR, Anchored’ OR ‘Anchored Polymerase Chain Reaction’ OR ’polymerase chain reaction’ |
| 2 | ‘Legionella’ OR ’legionella species’ OR ‘legionellae’ |
| 3 | 1 and 2 |

*Date of search: February 2, 2021
