# Supplementary material for: Detection performance of PCR for Legionella pneumophila in environmental samples: a systematic review and meta-analysis
Source: Ann Clin Microbiol Antimicrob. 2022 Mar 18;21:12. doi: 10.1186/s12941-022-00503-9 (PMC8934000; doi:10.1186/s12941-022-00503-9)
Supplement: Supplementary file 2 — Additional file 2: Figure S1. Flow diagram of inclusion and exclusion. [file 12941_2022_503_MOESM2_ESM.docx]

**Identification of studies via databases and registers**

Records removed *before screening*:

Duplicate records removed (n = 4079)

Records marked as ineligible by automation tools (n = 0)

Records removed for other reasons (n = 0)

Records identified from:

Databases (n = 7951)

Registers (n = 0)

**Identification**

Records screened

(n = 3872)

Records excluded manually

(n = 3809)

Reports sought for retrieval

(n = 63)

Reports not retrieved

(n = 0)

**Screening**

Reports excluded:

gold standard without culture (n = 11)

Inappropriate article type (n = 3)

Unable to construct 2×2 table (n = 21)

Sample size < 20 (n = 2)

Legionella spp. only (n = 8)

Other languages (n = 2)

Reports assessed for eligibility

(n = 16)

Studies included in review

(n = 16)

Reports of included studies

(n = 18)

**Included**

*From:*  Page MJ, McKenzie JE, Bossuyt PM, Boutron I, Hoffmann TC, Mulrow CD, et al. The PRISMA 2020 statement: an updated guideline for reporting systematic reviews. BMJ 2021;372:n71. doi: 10.1136/bmj.n71

For more information, visit: <http://www.prisma-statement.org/>
