# Supplementary material for: Detection performance of PCR for Legionella pneumophila in environmental samples: a systematic review and meta-analysis
Source: Ann Clin Microbiol Antimicrob. 2022 Mar 18;21:12. doi: 10.1186/s12941-022-00503-9 (PMC8934000; doi:10.1186/s12941-022-00503-9)
Supplement: Supplementary file 3 — Additional file 3: Table S2. Analysis of diagnostic threshold. [file 12941_2022_503_MOESM3_ESM.docx]

**Additional file 3. Analysis of Diagnostic Threshold**

| Var Coeff. Std. Error T p-value |
| --- |
| a 1.947 0.853 2.283 0.0364 |
| b( 1) 0.587 0.384 1.529 0.1459 |

Spearman correlation coefficient: -0.446 p-value= 0.064
